# Diagnostic Performance of Multimodal Large Language Models for Central Venous Catheter Assessment Chest Radiographs in the Intensive Care Unit

**DOI:** 10.3390/medsci14020315

**Published:** 2026-06-14

**Authors:** Christina-Chrysanthi Theocharidou, Zafeiris Tsinaris, Christos Karachristos, Anastasia Theocharidou, Michail Kourtidis, Kiriaki Papadopoulou, Athanasia-Marina Peristeri, Athanasios Astreinidis, Anna Simichanidou, Chrysavgi Giannaki, Myrto Tzimou, Evangelos Kaimakamis, Vasileios Voutsas, Vasiliki Soulountsi, Athina Lavrentieva

**Affiliations:** 1 1st Intensive Care Unit, General Hospital of Thessaloniki “G. Papanikolaou”, 57010 Thessaloniki, Greecevvoutsas@yahoo.gr (V.V.);; 2 Department of Microbiology, AHEPA University Hospital, School of Medicine, Aristotle University of Thessaloniki, 54636 Thessaloniki, Greece; 3 Radiology Department, Asklipios Diagnostic Center, 54622 Thessaloniki, Greece; 4 Interventional Radiology Unit, Clinical Radiology Department, AHEPA University Hospital, 54636 Thessaloniki, Greece; 5 Radiology Department, Theagenion Anti-Cancer Hospital, 54639 Thessaloniki, Greece; 6 Deparment of Medicine, Medical School, Democritus University of Thrace, 68100 Alexandroupoli, Greece

**Keywords:** multimodal large language models, chest radiography, central venous catheter, intensive care unit, diagnostic performance, pneumothorax

## Abstract

Background: Chest radiography remains central to post-procedural assessment of central venous catheter (CVC) placement in intensive care units. Multimodal large language models (MLLMs) can process medical images, but their reliability for practical radiography tasks remains uncertain. This study assessed the diagnostic performance of MLLMs and intensivists for CVC access classification, CVC tip assessment, and pneumothorax-related radiographic findings. Methods: In this retrospective diagnostic performance study, consecutive portable anteroposterior chest radiographs obtained after CVC placement in adult critically ill patients were independently evaluated by four intensivists and five MLLMs. A radiologist consensus served as the reference standard. Interobserver agreement and diagnostic performance were assessed using Fleiss’ kappa, Gwet AC1, Cohen’s kappa, accuracy, sensitivity, specificity, precision, F1 score, balanced accuracy, and Matthews correlation coefficient. Results: The final cohort included 183 unique radiographs. Intensivist reviewers showed high performance for CVC access classification but lower and more heterogeneous performance for CVC tip-position assessment. Among MLLMs, CVC access accuracy ranged from 0.339 to 0.874, whereas CVC tip assessment was dominated by almost universal classification of tips as appropriate, with near-zero specificity and chance-level balanced accuracy. For pneumothorax-related findings, all MLLMs classified every case as negative. Intensivist reviewers had higher balanced accuracy than MLLMs for CVC access classification (difference, 0.420; 95% CI, 0.349–0.490; *p* < 0.001) and CVC tip assessment (difference, 0.247; 95% CI, 0.205–0.290; *p* < 0.001). Pneumothorax analyses were exploratory because only five positive cases were present. Conclusions: The evaluated MLLMs showed unreliable diagnostic performance compared with experienced intensivists. Apparent performance was influenced by class imbalance and dominant-response behavior, supporting cautious task-specific validation and complete diagnostic performance reporting.

## 1. Introduction

Despite advances in imaging modalities and the increasing use of ultrasound, chest radiography remains the most frequently utilized imaging tool in intensive care units, particularly for the evaluation of central venous catheter (CVC) placement and related complications [1,2]. However, the manual interpretation of these radiographs imposes a substantial burden on clinical workflows [3,4,5]. Clinicians often rely on their own interpretation of portable anteroposterior chest radiographs to confirm appropriate catheter positioning and to exclude immediate post-procedural complications, such as pneumothorax [2].

Artificial intelligence (AI) may reduce this diagnostic burden by automating selected image-analysis tasks [6]. Computer vision applications in chest radiography have relied on supervised deep learning models, such as convolutional neural networks (CNNs) trained on labeled radiographic datasets for specific diagnostic targets [4,7,8,9]. In contrast, multimodal large language models (MLLMs) process visual and textual inputs jointly, enabling image evaluation through natural language prompting without task-specific retraining [6,9,10]. These models are widely accessible, raising the possibility of informal clinical use. However, their prompt-based application is not grounded in specialized radiographic training, and reported performance remains highly inconsistent across studies [10,11,12,13,14,15].

Evaluating these systems under clinical conditions is necessary to determine their diagnostic capabilities. We hypothesized that because prompted MLLMs lack explicit training in radiographic geometry and localized anatomical structures, their diagnostic performance on critical care chest radiographs would be inferior to that of experienced clinicians. The primary objective of this study was to compare the diagnostic performance of five MLLMs against four intensivists, using a three-radiologist consensus as the reference standard. We evaluated three tasks: (a) CVC access site classification, (b) CVC tip position assessment, and (c) detection of findings related to pneumothorax. Our secondary objectives were to compare group-level performance and assess the impact of image quality.

## 2. Materials and Methods

### 2.1. Study Design

We conducted a retrospective observational diagnostic performance study at a tertiary care academic hospital. The study population comprised adult critically ill patients who underwent jugular or subclavian CVC placement between 1 January 2024 and 31 December 2024 and had a post-procedural chest radiograph available for review. The institutional review board approved the study protocol (65/23.01.2025) before data collection, and the requirement for informed consent was waived because of the retrospective design and use of de-identified data. A preliminary analysis using a subset of the present dataset was presented at a scientific conference in 2025 [16].

### 2.2. Study Population and Case Selection

All consecutive chest radiographs obtained after CVC placement in intensive care unit patients during the study period were screened. Radiographs were eligible if they were obtained from patients aged 18 years or older, corresponded to an intensive care unit admission, were performed after CVC insertion, and represented a unique catheter-placement episode. When more than one radiograph was available for the same placement episode, only the earliest image was retained.

We excluded duplicate radiographs from the same placement episode, as well as cases where the catheter was incompletely visualized (because of extremely low image quality or suboptimal positioning) or where two ipsilateral CVCs precluded unambiguous assessment of catheter course or tip position (e.g., concurrent ipsilateral jugular and subclavian catheters). Because the study used a fixed retrospective consecutive cohort, no formal a priori sample-size calculation was performed.

### 2.3. Image Acquisition

Portable anteroposterior chest radiographs were retrospectively retrieved from the electronic health record and exported as de-identified JPEG image files. All images had fixed dimensions of 2828 × 2320 pixels and were stored in 8-bit RGB format. Visible patient identifiers were removed before analysis, and no other image editing was applied. The same de-identified JPEG files were provided to all assessors. Each image was assigned a de-identified study code, consisting of coded indicators of patient sex (male or female) and catheter laterality (right or left), followed by a randomized numeric identifier (e.g., FR001, ML002).

### 2.4. Human Reviewers and Reference Standard

Four intensivists (R1–R4), each with at least 10 years of clinical experience, independently reviewed all radiographs. Three radiologists, each with more than 5 years of experience, independently reviewed all images. Discrepancies between radiologists were resolved by consensus, and the final consensus assessment was designated as the reference standard (R5).

All reviewers were provided with details of the catheter laterality (right or left) and patient sex (male or female). Intensivist reviewers were blinded to the R5 consensus reference annotations, the responses of other intensivist reviewers, and all MLLM outputs. Responses were recorded using a locally hosted HTML-based interface stored on a USB flash drive to ensure completeness, blinding, and reviewer anonymity. Consensus among the intensivist reviewers was not used as the ground truth because the objective was to evaluate both intensivist and MLLM performance against an expert radiologist consensus reference standard.

### 2.5. Multimodal Large Language Model Evaluation

Five MLLMs were evaluated between 7 and 30 January 2026: Gemini 3 Flash (Google LLC, Mountain View, CA, USA), GPT-5.1 (OpenAI, San Fransisco, CA, USA), Grok 4.1 (xAI, Palo Alto, CA, USA), Claude Opus 4 (Anthropic, San Fransisco, CA, USA), and MedGemma 1.5 (Google Deepmind, London, UK) [10]. Gemini 3 Flash, GPT-5.1, Grok 4.1, and Claude Opus 4 were accessed through the “OpenRouter” platform, whereas MedGemma 1.5 was executed in “Google Colab”. Exact model identifiers, access routes, evaluation dates, and settings are provided in Table S1; the shared prompt template is provided in Table S2. Each model evaluated each image independently and was provided with the same contextual information available to human reviewers, namely, catheter laterality and patient sex. Model assessment was performed using a standardized Python script (Python 3.13.1). Visual inputs were encoded as base64 strings and transmitted at their native resolution of 2828 × 2320 pixels without any pre-processing, resizing, or compression. For each image, a new HTTPS session was initiated, and no conversation history was retained, ensuring independent assessment and preventing carryover from prior cases. Image order and categorical response option order were randomized to reduce ordering bias. For each evaluation, the processing time per image was recorded. The full evaluation logic and details of session management are documented via structured pseudocode (Table S3) and a schematic flowchart (Figure S1) in Supplementary Material S1.

### 2.6. Clinical Tasks and Outcome Definitions

For each radiograph, human reviewers and MLLMs answered the same three prespecified clinical questions. The first task classified the catheter access site as jugular or subclavian. The second task assessed whether the CVC tip position was appropriate. The third task assessed whether the radiograph showed findings suggestive of pneumothorax. CVC access site classification and CVC tip position assessment were the primary tasks. Detection of findings suggestive of pneumothorax was a predefined exploratory task, as positive cases were expected to be uncommon. All the radiographs included contained a visible CVC on the predefined side.

Appropriate catheter tip position was defined as the projection of the catheter tip within the lower third of the superior vena cava, the cavoatrial junction, or the high right atrium [17]. Pneumothorax was assessed as a binary outcome based on the presence of radiographic findings suggestive of pneumothorax, rather than a definitive clinical diagnosis. These findings included a visible pleural line, absence of peripheral lung markings, increased hemithoracic lucency, partial or complete lung collapse, deep sulcus sign, and subcutaneous emphysema. Because such findings were expected to be uncommon in the dataset, analyses for this task were considered exploratory and interpreted cautiously.

### 2.7. Data Extraction and Management

Reviewer and model responses were entered into a structured spreadsheet. For each image, the dataset included a unique image identifier, catheter laterality, patient sex, reviewer or model identifier, access site classification, catheter tip assessment, and pneumothorax assessment. Before analysis, the dataset was reviewed for completeness, consistency, and valid response coding. No discrepancies requiring correction were identified.

### 2.8. Statistical Analysis

Case characteristics and response distributions were summarized using counts and percentages. Interobserver agreement was evaluated separately for human reviewers and MLLMs. Agreement was calculated for each task and across tasks using Fleiss’ kappa and Gwet AC1. Both metrics were reported because kappa can be affected by class imbalance and prevalence effects, whereas Gwet AC1 provides a complementary estimate of corrected agreement under such conditions. Diagnostic performance for each human reviewer and each model was evaluated against the radiologist consensus reference standard, using primarily Cohen’s kappa. Secondary performance metrics were accuracy, sensitivity, specificity, precision, F1 score, balanced accuracy, and Matthews correlation coefficient (MCC). To compare group-level performance between human reviewers and MLLMs, balanced accuracy was used as the primary metric and was compared using a case-resampling bootstrap with 2000 replicates. In each replicate, cases were sampled with replacement, group-level mean performance was recalculated, and the difference between human and model performance was estimated as the human mean minus the model mean. Mean Cohen’s kappa, accuracy, sensitivity, specificity, precision, F1 score, and MCC were reported as secondary metrics using the same approach. Analyses were performed using custom Python scripts in Python 3.13.1, with pandas 2.2.3 and NumPy 2.2.4.

Difficult-case analyses were performed to characterize potential ambiguity and systematic failure patterns. A case was classified as human-difficult if at least three of the four intensivist reviewers disagreed with the radiologist consensus reference standard. A case was classified as model-difficult if at least three of the five multimodal large language models disagreed with the consensus reference standard. Cases were then categorized as human-difficult only, model-difficult only, or shared difficult. To distinguish group-specific errors from intrinsically ambiguous cases, primary analyses were repeated after excluding shared difficult cases.

### 2.9. Radiograph Quality

The radiologist consensus panel assessed radiographs’ technical quality using four prespecified criteria: adequate penetration/exposure, adequate inspiration, absence of substantial patient angulation, and absence of substantial rotation [13]. One point was assigned for each criterion met. Radiographs with scores of 3 or 4 were classified as good quality, and those with scores of 0 to 2 were classified as poor quality. Agreement and performance analyses were repeated within each quality stratum. Quality-stratified analyses were summarized descriptively using mean balanced accuracy between good- and poor-quality radiographs within each assessor group.

## 3. Results

### 3.1. Study Cohort and Case Characteristics

**Figure 1:**
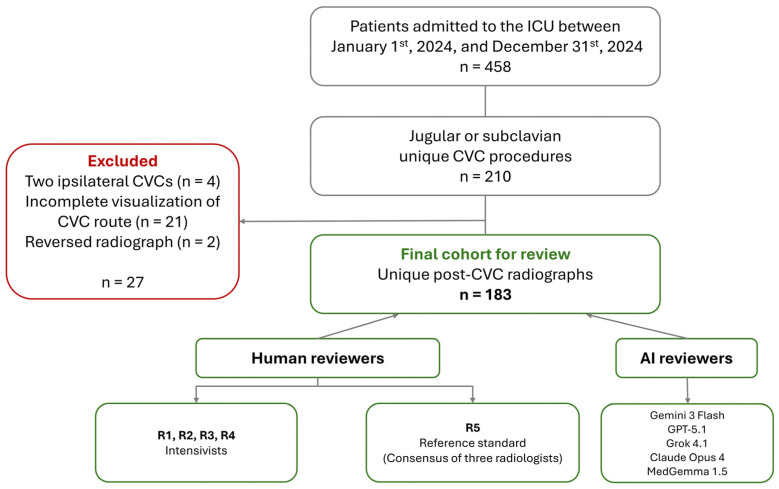
Study cohort derivation and radiograph assessment workflow. AI = artificial intelligence; CVC = central venous catheter; ICU = intensive care unit.

The final analytic cohort included 183 unique post-procedural portable anteroposterior chest radiographs obtained after CVC placement (Figure 1). Cohort characteristics, catheter laterality and access site distributions, tip position assessment, and findings related to pneumothorax are summarized in Table 1. Representative examples of the chest radiographs evaluated in this study, along with a detailed comparison of human and MLLM evaluations, are provided in Supplementary Material S2. Mean ± standard deviation processing times per image were 6.2 ± 0.8 s for Gemini 3 Flash, 7.6 ± 0.9 s for MedGemma 1.5, 16.9 ± 1.0 s for Claude Opus 4, and 19.3 ± 3.9 s for GPT-5.1, while the median processing time was 9.6 s [IQR: 8.6–10.8 s] for Grok 4.1-fast.

### 3.2. Response Distributions

Human reviewer response distributions were broadly similar to the reference standard for CVC access classification. Across the four intensivists, jugular access was selected in 86.3% to 90.7% of cases. Greater variability was observed for catheter tip assessment: the proportion of cases classifying tip position as appropriate ranged from 55.7% to 85.2%. The presence of findings related to pneumothorax was recorded in responses in 2.7% to 4.4% of cases.

MLLM response distributions differed substantially across models and tasks. For CVC access, the proportion of jugular classifications ranged from 24.0% for MedGemma 1.5 to 94.0% for Gemini 3 Flash. For the CVC tip position, three models classified all 183 cases as appropriate, while Gemini 3 Flash and GPT-5.1 classified 98.4% and 97.8% of cases as appropriate, respectively. For pneumothorax, all five models classified all 183 cases as negative.

### 3.3. Interobserver Agreement

Agreement among intensivist reviewers was highest for CVC access classification and lower for CVC tip-position assessment. For pneumothorax-related findings, the discrepancy between Fleiss’s kappa and Gwet AC1 reflected the highly imbalanced response distribution. Among MLLMs, agreement patterns were driven largely by uniform or near-uniform responses, including near-universal classification of catheter tips as appropriate and universal classification of pneumothorax-related findings as absent. Full agreement estimates for each task and assessor group are summarized in Table 2.

### 3.4. Diagnostic Performance Relative to the Radiologist Consensus Reference Standard

Individual intensivist reviewer and model diagnostic performance estimates are provided in Table S4, and corresponding confusion matrix counts are provided in Table S5. Intensivist reviewers showed consistently high performance for CVC access classification. Accuracy ranged from 0.962 to 0.995, balanced accuracy from 0.854 to 0.997, Cohen’s kappa from 0.808 to 0.976, and MCC from 0.824 to 0.977. Human performance was lower and more heterogeneous for CVC tip-position assessment, with accuracy ranging from 0.781 to 0.814, balanced accuracy from 0.668 to 0.846, Cohen’s kappa from 0.392 to 0.611, and MCC from 0.433 to 0.640. For findings suggestive of pneumothorax, intensivist reviewers had high raw accuracy because most cases were negative, but sensitivity, precision, F1 score, and MCC were unstable because only five reference-standard positive cases were present.

MLLM performance differed substantially by task. For CVC access classification, model accuracy ranged from 0.339 for MedGemma 1.5 to 0.874 for Gemini 3 Flash, and Cohen’s kappa against the reference standard ranged from −0.018 for Grok 4.1 to 0.284 for Gemini 3 Flash. For CVC tip assessment, MLLM raw accuracy ranged from 0.689 to 0.700, but this was driven by an almost universal prediction of appropriate tip position. Model sensitivity ranged from 0.977 to 1.000, while specificity ranged from 0.000 to 0.018; balanced accuracy was therefore approximately chance level for all models. For pneumothorax-related findings, all five MLLMs classified every case as negative, yielding a specificity of 1.000 but a sensitivity of zero and balanced accuracy of 0.500 for each model. Regarding Cohen’s kappa against the reference standard (R5), in CVC access classification, Gemini 3 Flash ranked highest among the MLLMs (κ = 0.284), followed by MedGemma 1.5 (κ = 0.044), GPT-5.1 (κ = 0.009), Claude Opus 4 (κ = −0.006), and Grok 4.1 (κ = −0.018). For CVC tip assessment, all MLLM kappa values were close to zero, ranging from −0.007 to 0.004, indicating chance-level agreement with the radiologist consensus reference standard. For pneumothorax-related findings, all five MLLMs also had a kappa equal to zero.

### 3.5. Group-Level Comparison of Human Intensivist Reviewers and MLLMs

Group-level comparisons are summarized in Table 3. For CVC access classification, human reviewers had higher mean values than MLLMs across all evaluated diagnostic performance metrics, including accuracy, sensitivity, specificity, balanced accuracy, Cohen’s kappa, and MCC. For CVC tip assessment, human reviewers had higher mean accuracy, specificity, precision, balanced accuracy, Cohen’s kappa, and MCC. MLLMs had higher mean sensitivity, consistent with their near-universal classification of catheter tips as appropriate, while the group difference in F1 score was not statistically significant. For pneumothorax-related findings, the mean raw accuracy was similar between groups. Intensivist reviewers had higher mean sensitivity, balanced accuracy, and Cohen’s kappa, whereas MLLMs had higher specificity because all model responses were negative for this task. Precision, F1 score, and MCC differences were not estimable for pneumothorax-related findings because all MLLMs classified all cases as negative. Given the small number of positive cases, confidence intervals and *p*-values for this task are unstable and should not be interpreted as precise estimates.

### 3.6. Difficult Cases and Systematic Error Patterns

MLLMs had substantially more difficult cases than human reviewers for CVC access and CVC tip-position assessment. For CVC access, no case met the human-difficult definition, whereas 63 cases were MLLM-difficult. All MLLMs were wrong in 2 cases. For CVC tip assessment, 19 cases were difficult for human reviewers, and 55 were difficult for MLLMs. Thirteen cases were difficult for both groups, six were human-only difficult cases, and forty-two were MLLM-only difficult cases. All four human reviewers were wrong in 2 cases, whereas all five MLLMs were wrong in 53 cases. For findings related to pneumothorax, two cases were difficult for human reviewers, and five cases were difficult for MLLMs. Two cases were difficult for both groups, and three were MLLM-only difficult cases. All five MLLMs missed all five positive pneumothorax-related cases.

After excluding shared-difficult cases, analysis was performed only for CVC tip-position assessment, because no shared-difficult cases were identified for CVC access classification, and pneumothorax-related findings included only five reference standard positive cases. After removal of 13 shared-difficult CVC tip cases, 170 cases remained; intensivist reviewers continued to outperform MLLMs, and MLLMs continued to show near-chance balanced accuracy driven by near-universal classification of catheter tips as appropriate (Table S6).

False positive and false negative counts showed task-specific model error patterns. For CVC access classification, error direction varied across models, with some models more often misclassifying jugular catheters as subclavian and others more often misclassifying subclavian catheters as jugular. For CVC tip assessment, errors were more consistent: all models showed a strong false positive pattern, frequently classifying inappropriate catheter tip positions as appropriate. This was most pronounced in Grok 4.1, MedGemma 1.5, and Claude Opus 4, each of which produced 55 false positives and no false negative tip position errors. For pneumothorax-related findings, all five MLLMs classified every case as negative, resulting in no false positive errors but five false negative errors for each model.

### 3.7. Quality-Stratified Analysis

Descriptive quality-stratified analyses, summarized as mean balanced accuracy within each task and assessor group, are presented in Table S7. For CVC access, mean human balanced accuracy was 0.946 in good-quality radiographs and 0.964 in poor-quality radiographs, compared with MLLM values of 0.538 and 0.519, respectively. For CVC tip-position assessment, mean human balanced accuracy was 0.748 in good-quality radiographs and 0.749 in poor-quality radiographs, compared with MLLM values of 0.499 and 0.502. For pneumothorax-related findings, mean human balanced accuracy was 0.773 in good-quality radiographs and 0.986 in poor-quality radiographs, while MLLM balanced accuracy was 0.500 in both strata. Pneumothorax quality-stratified analyses are exploratory and should be interpreted cautiously because positive cases were sparse after stratification.

## 4. Discussion

This retrospective diagnostic performance study found that experienced intensivists consistently outperformed contemporary MLLMs in evaluating chest radiographs for CVC placement and complications. The separation between humans and MLLMs was most apparent when performance was judged using metrics that account for class imbalance and error direction. For CVC access classification, human reviewers had high accuracy, balanced accuracy, Cohen’s kappa, and MCC, whereas the best performing MLLM remained below the lowest performing human reviewer. For CVC tip assessment, MLLM raw accuracy was misleading because models overwhelmingly classified tips as appropriate. For pneumothorax-related findings, all MLLMs classified all cases as negative, producing an apparently high accuracy but with a sensitivity equal to zero.

Agreement results require cautious interpretation because several tasks were affected by class imbalance and near-uniform response patterns. For CVC tip assessment, MLLMs had a Fleiss’ kappa of −0.008 but a Gwet AC1 of 0.985. This divergence should not be interpreted as clinically meaningful agreement; rather, it reflects the models’ tendency to give the same positive response for almost every case. Similarly, pooled MLLM agreement across all tasks, Fleiss’ kappa 0.764 and Gwet AC1 0.777, was strongly influenced by uniform responses in imbalanced tasks, particularly near-universal positive classification for CVC tip position and universal negative classification for pneumothorax-related findings. These findings show why agreement statistics should be interpreted alongside response distributions and confusion matrix counts.

The central finding is not simply that the models made more errors, but that their errors were systematic and task dependent. For pneumothorax-related findings, the complete absence of positive model predictions suggests a strong dominant class tendency in a rare-outcome setting. However, MLLM failure was not limited to rare-event detection. The models also performed poorly on CVC access classification, a task that did not contain rare events. This suggests a broader limitation in radiographic line or tube localization and specific anatomical discrimination. For CVC tip assessment, the models converged on a clinically problematic default response: nearly all catheter tips were labeled appropriate, causing specificity to collapse despite high sensitivity. Together, these patterns indicate that raw accuracy alone can substantially overstate model competence and that MLLM errors may arise from both prevalence-driven answers and a failure of spatial radiographic reasoning. Finally, although the rapid processing times of these models (averaging under 20 s per image) might suggest feasibility in fast-paced ICU workflows, speed does not translate to clinical utility when diagnostic performance remains poor, and error patterns are systematic.

These systematic errors highlight the distinction between a model’s capacity to receive visual input and its diagnostic accuracy. Rather than mapping precise anatomical coordinates or tracing physical lines, these models process visual patterns to generate text descriptions. Consequently, while they can recognize a chest radiograph and generate a general description, they lack the capacity to follow a catheter’s path, evaluate the position of a tip relative to landmarks, or detect faint density changes. Unlike supervised architectures trained to localize objects, prompted MLLMs map whole images directly to text, leaving them vulnerable to relying on highly probable default clinical assumptions rather than structural evidence in the image.

These results have practical implications for evaluating MLLMs in radiology workflows. Reporting accuracy alone, or a limited set of aggregate metrics, can obscure clinically important failures. In this study, apparently reasonable raw accuracy for CVC tip assessment concealed the near total failure to identify inappropriate tip positions, and high pneumothorax accuracy concealed complete failure to detect positive cases. Validation studies of MLLMs for medical imaging should therefore report all metrics: sensitivity, specificity, balanced accuracy, Cohen’s kappa, MCC, and full confusion matrix counts. Additionally, to address the statistical challenges of rare complications like pneumothorax, future validation studies should explore alternative methodologies. A consecutive cohort is necessary to represent real-world prevalence for primary CVC placement tasks. However, future studies focused on rare complications should employ stratified sampling or case–control enrichment to oversample positive cases, pool data via multi-center collaborations, or extend patient accrual periods to achieve more balanced sample sizes.

Prior studies have reported variable performance of MLLMs in radiographic interpretation. In a study focused on pneumothorax, GPT-4o showed relatively high specificity but lower sensitivity, with performance limitations for small pneumothoraces [14]. In trauma radiographs, GPT-4o performed substantially worse than emergency medicine and orthopedic specialists, supporting concerns that general-purpose models may struggle with clinically important radiographic abnormalities [11]. In hand radiographs, GPT-based models showed only modest agreement for growth stage classification, again suggesting that broad image reasoning does not necessarily translate into reliable radiographic classification [12]. Conversely, Kim and Kim found that diagnostic performance for radiolucent jaw lesions improved when models were given richer multimodal inputs and structured multiple-choice formats, indicating that task framing and input context can materially influence performance [13]. Differences in pneumothorax prevalence, image type, and task framing may explain part of the discrepancy between the focused pneumothorax results reported by Akçay et al. [14] and the universal negative pneumothorax predictions in our study. However, these factors do not explain the poor MLLM performance observed for CVC access classification, suggesting that current MLLMs also struggle with the spatial and anatomical reasoning required for this assessment. The present study extends prior work by evaluating five MLLMs on pragmatic post-procedural chest radiographs in critically ill patients and aligns with studies demonstrating that the MLLM visual capabilities still have systematic shortcomings [11,15].

Several features strengthen these findings. Intensivist reviewers and five MLLMs were evaluated against the same three-radiologist consensus reference standard under the same conditions. All assessors reviewed the same de-identified clinically exported image files with the same contextual variables, supporting the internal fairness of the comparison. The cohort used real radiographs from critically ill patients rather than idealized teaching images, making the task closer to the practical environment of post-procedural catheter assessment. The model evaluation minimized conversational carryover by using a fresh independent session for each image, with no retained conversation history. Finally, the analysis reported a broad set of diagnostic metrics, agreement statistics, difficult-case analyses, quality-stratified summaries, and individual reviewer rankings, which is particularly important in imbalanced datasets.

This study has limitations. Firstly, it was conducted at a single center using a fixed retrospective consecutive cohort, which may limit generalizability to other institutions, image acquisition protocols, patient populations, and catheter types. Secondly, because all eligible cases from the predefined study period were included, no formal a priori sample size calculation was performed. As a result, analyses of uncommon outcomes, particularly pneumothorax-related findings, were limited by the number of positive cases. The pneumothorax-related outcome included only 5 positive cases; metrics calculated for this task are therefore exploratory and imprecise. Furthermore, inputs were de-identified JPEG files exported from the clinical workflow rather than raw DICOM radiographs. This limits image-input fidelity and workflow generalizability, but not the internal comparison, because human reviewers and MLLMs evaluated identical files under identical image conditions. Moreover, the evaluated MLLMs were tested through the access routes and model versions available in January 2026, and model behavior may change as systems are constantly updated. Additionally, the study assessed categorical outputs from static radiographs and did not evaluate integration with clinical history, serial imaging, bedside ultrasound, raw DICOM metadata, or prospective clinician notes. Finally, it should also be noted that the present study, like all current MLLM evaluations, cannot verify whether model outputs were truly grounded in the radiographic content. MLLMs may generate contextually plausible responses based on prompt context, such as that of a post-CVC radiograph, rather than through genuine visual recognition. The systematic failure patterns observed in this study, particularly the universal negative classification for pneumothorax and near-universal classification of CVC tips as appropriate, are themselves consistent with dominant-response behavior rather than visual recognition. A potential mitigation would be to incorporate a preliminary grounding question, such as confirming CVC visibility before proceeding to classification. However, this does not resolve the limitation: the model can just as confidently hallucinate a “yes” answer to the preliminary question as it can hallucinate the diagnostic answer itself. A hallucinating model can pass the triage check and then proceed to hallucinate the clinical classification.

## 5. Conclusions

In post-procedural chest radiographs after CVC placement in critically ill patients, the evaluated MLLMs showed unreliable diagnostic performance compared with experienced intensivists. Apparent performance was often inflated by class imbalance and dominant-response behavior, particularly for CVC tip assessment and detection of findings related to pneumothorax. These findings support the cautious, task-specific validation of MLLMs before clinical use of their vision capabilities and emphasize that clinical AI evaluation should prioritize complete diagnostic performance reporting rather than raw accuracy alone.

## Figures and Tables

**Table 1:** Cohort characteristics according to the reference standard.

Characteristic	Value
Total radiographs	183
Male sex	98 (53.6%)
Female sex	85 (46.4%)
Right-sided catheter	120 (65.6%)
Left-sided catheter	63 (34.4%)
Jugular access	159 (86.9%)
Subclavian access	24 (13.1%)
Appropriate CVC tip position	128 (69.9%)
Inappropriate CVC tip position	55 (30.1%)
Pneumothorax-related findings absent	178 (97.3%)
Pneumothorax-related findings present	5 (2.7%)

CVC = central venous catheter.

**Table 2:** Interobserver agreement among intensivist reviewers and MLLMs.

Task	Group	Fleiss’ Kappa (95% CI)	Gwet AC1 (95% CI)
CVC access	Human	0.868 (0.779 to 0.933)	0.964 (0.937 to 0.983)
CVC position	Human	0.398 (0.294 to 0.496)	0.597 (0.510 to 0.675)
Pneumothorax	Human	0.449 (0.029 to 0.692)	0.958 (0.933 to 0.979)
Overall	Human	0.848 (0.820 to 0.873)	0.867 (0.843 to 0.889)
CVC access	MLLM	−0.020 (−0.060 to 0.020)	0.042 (−0.005 to 0.099)
CVC position	MLLM	−0.008 (−0.014 to −0.003)	0.985 (0.971 to 0.993)
Pneumothorax	MLLM	1.000 (1.000 to 1.000)	1.000 (1.000 to 1.000)
Overall	MLLM	0.764 (0.739 to 0.789)	0.777 (0.749 to 0.805)

Values are agreement estimates with 95% CIs in parentheses. MLLM = multimodal large language model.

**Table 3:** Group-level intensivists vs. MLLMs diagnostic performance comparisons.

Task	Metric	Intensivists Minus MLLMs Difference	95% Bootstrap CI	*p* Value
CVC access	Cohen’s kappa	0.865	0.775 to 0.957	<0.001
CVC access	Accuracy	0.405	0.373 to 0.439	<0.001
CVC access	Sensitivity	0.399	0.369 to 0.433	<0.001
CVC access	Specificity	0.440	0.300 to 0.575	<0.001
CVC access	Precision	0.101	0.056 to 0.151	<0.001
CVC access	F1-score	0.305	0.276 to 0.339	<0.001
CVC access	Balanced accuracy	0.420	0.349 to 0.490	<0.001
CVC access	MCC	0.853	0.752 to 0.963	<0.001
CVC tip position	Cohen’s kappa	0.497	0.404 to 0.584	<0.001
CVC tip position	Accuracy	0.100	0.036 to 0.161	0.001
CVC tip position	Sensitivity	−0.121	−0.160 to −0.085	<0.001
CVC tip position	Specificity	0.616	0.540 to 0.692	<0.001
CVC tip position	Precision	0.155	0.116 to 0.194	<0.001
CVC tip position	F1-score	0.036	−0.002 to 0.072	0.061
CVC tip position	Balanced accuracy	0.247	0.205 to 0.290	<0.001
CVC tip position	MCC	0.526	0.382 to 0.652	<0.001
Pneumothorax-related findings	Cohen’s kappa	0.541	0.076 to 0.795	0.017
Pneumothorax-related findings	Accuracy	−0.001	−0.022 to 0.022	0.914
Pneumothorax-related findings	Sensitivity	0.650	0.154 to 1.000	0.017
Pneumothorax-related findings	Specificity	−0.020	−0.031 to −0.010	<0.001
Pneumothorax-related findings	Precision	N/A	N/A	N/A
Pneumothorax-related findings	F1-score	N/A	N/A	N/A
Pneumothorax-related findings	Balanced accuracy	0.315	0.067 to 0.492	0.017
Pneumothorax-related findings	MCC	N/A	N/A	N/A

Differences are intensivist minus MLLM mean performance. Confidence intervals and *p* values were estimated using case-resampling bootstrap with 2000 replicates. Pneumothorax-related analyses are exploratory because only 5 reference standard positive cases are present; confidence intervals and *p*-values for this task are unstable and should not be interpreted as precise estimates. N/A indicates not estimable. MLLM = multimodal large language model; CVC = central venous catheter.

## Data Availability

The raw clinical data and chest radiographs evaluated in this study are available upon reasonable request from the corresponding author. The data are not publicly archived due to patient privacy protection and confidentiality regulations under the approved institutional ethical framework (General Hospital of Thessaloniki ‘G. Papanikolaou’).

## Supplementary Materials

The following supporting information can be downloaded at https://www.mdpi.com/article/10.3390/medsci14020315/s1: Supplementary Material S1: Table S1: model identifiers, access routes, evaluation dates, and settings; Table S2: Prompt template used for MLLM evaluation; Figure S1: Schematic of the image evaluation and session management pipeline; Table S3: Pseudocode of the image evaluation and prompt randomization script; Table S4: Diagnostic performance by individual intensivist reviewer or model; Table S5: Confusion matrix counts for intensivist reviewers and models; Table S6: Group-level diagnostic performance after excluding shared-difficult cases; Table S7: Quality-stratified mean balanced accuracy by task and assessor group. Supplementary Material S2: Examples of Radiograph Cases with Responses.

## Abbreviations

The following abbreviations are used in this manuscript:

CI	Confidence interval
CVC	Central venous catheter
DICOM	Digital Imaging and Communications in Medicine
HTML	HyperText Markup Language
HTTPS	HyperText Transfer Protocol Secure
ICU	Intensive care unit
MCC	Mathews correlation coefficient
MLLM	Multimodal large language model
RGB	Red–green–blue
USB	Universal Serial Bus

## References

[1] Toy D., Siegel M.D., Rubinowitz A.N. (2022). Imaging in the Intensive Care Unit. Semin. Respir. Crit. Care Med..

[2] Villasana-Gomez G., Toussie D., Kaufman B., Stojanovska J., Moore W.H., Azour L., Traube L., Ko J.P. (2024). Chest Intensive Care Unit Imaging. Clin. Chest Med..

[3] Khair M.A.W., Alamri S.M., Alrashidi A.H., Lharbi M.A., Al-Moghamsi Y.S., Alharbi M.M. (2021). The Role of Chest X-Ray in Intensive Care Unit. Int. J. Innov. Res. Med. Sci..

[4] Tanaka K., Nakada T., Takahashi N., Dozono T., Yoshimura Y., Yokota H., Horikoshi T., Nakaguchi T., Shinozaki K. (2021). Superiority of Supervised Machine Learning on Reading Chest X-Rays in Intensive Care Units. Front. Med..

[5] Rachh P., Levey A.O., Lemmon A., Marinescu A., Auffermann W.F., Haycook D., Berkowitz E.A. (2018). Reducing STAT Portable Chest Radiograph Turnaround Times: A Pilot Study. Curr. Probl. Diagn. Radiol..

[6] Yuan M., Bao P., Yuan J., Shen Y., Chen Z., Xie Y., Zhao J., Li Q., Chen Y., Zhang L. (2024). Large Language Models Illuminate a Progressive Pathway to Artificial Intelligent Healthcare Assistant. Med. Plus.

[7] Rajpurkar P., Irvin J., Zhu K., Yang B., Mehta H., Duan T., Ding D., Bagul A., Langlotz C., Shpanskaya K. (2017). CheXNet: Radiologist-Level Pneumonia Detection on Chest X-Rays with Deep Learning 2017. arXiv.

[8] Ronneberger O., Fischer P., Brox T. (2015). U-Net: Convolutional Networks for Biomedical Image Segmentation. Proceedings of the International Conference on Medical Image Computing and Computer-Assisted Intervention, Munich, Germany, 5–9 October 2015.

[9] Dutta N., Bose K., Syailendra E., Chu L., Gupta P. (2026). Vision-Language Models in Diagnostic Imaging: Review of Technical Advances, Clinical Validation, and Practical Deployment. Int. J. Med. Inform..

[10] Sellergren A., Kazemzadeh S., Jaroensri T., Kiraly A., Traverse M., Kohlberger T., Xu S., Jamil F., Hughes C., Lau C. (2025). MedGemma Technical Report. arXiv.

[11] Öztürk A., Günay S., Ateş S., Yiğit Y. (2025). Can Gpt-4o Accurately Diagnose Trauma X-Rays? A Comparative Study with Expert Evaluations. J. Emerg. Med..

[12] Yıldırım A., Cicek O., Genç Y.S. (2025). Can AI-Based ChatGPT Models Accurately Analyze Hand–Wrist Radiographs? A Comparative Study. Diagnostics.

[13] Kim K., Kim B.C. (2025). Diagnostic Performance of Large Language Models in Multimodal Analysis of Radiolucent Jaw Lesions. Int. Dent. J..

[14] Akçay O., Özel A., Öztürk Ö., Acar T., Tekneci A.K., Akçam T.İ., Gürsoy S. (2025). Evaluation of the Effectiveness of the ChatGPT Artificial Intelligence Application in the Diagnosis of Spontaneous Pneumothorax on Chest Radiograph Interpretation. BMC Pulm. Med..

[15] Tong S., Liu Z., Zhai Y., Ma Y., LeCun Y., Xie S. (2024). Eyes Wide Shut? Exploring the Visual Shortcomings of Multimodal LLMs. Proceedings of the 2024 IEEE/CVF Conference on Computer Vision and Pattern Recognition (CVPR).

[16] Theocharidou C.C., Tsinaris Z., Karachristos C., Theocharidou A., Kourtidis M., Papadopoulou K., Giannaki C., Kaimakamis E., Papaioannou M., Voutsas V. Reliability of Multimodal Large Language Models in Evaluating Correct Placement of Central Venous Catheters in Critically Ill Patients. ESICM LIVES 2025, Proceedings of the European Society of Intensive Care Medicine Annual Congress.

[17] Johnston A.J., Simpson M.J., McCormack V., Barton A., Bennett J., Chalisey A., Crane J., Curry S., Laycock H., Patel D. (2025). Association of Anaesthetists Guidelines: Safe Vascular Access 2025. Anaesthesia.

