# Supplementary material for: Diagnostic Performance of Multimodal Large Language Models for Central Venous Catheter Assessment Chest Radiographs in the Intensive Care Unit
Source: Med Sci (Basel). 2026 Jun 14;14(2):315. doi: 10.3390/medsci14020315 (PMC13302893; doi:10.3390/medsci14020315)
Supplement: Supplementary file 1 [file medsci-14-00315-s001.zip › Supplementary_Material_S1.pdf]

Supplementary Material S1

Title

Diagnostic Performance of Multimodal Large Language Models for Central Venous Catheter Assessment Chest Radiographs in the Intensive Care Unit

Index of Supplementary Material

	Title
Table S1	Model Identifiers, Access Routes, Evaluation Dates, and Settings
Table S2	Prompt Template Used for MLLM Evaluation
Figure S1	Schematic of the image evaluation and session management pipeline.
Table S3	Pseudocode of the image evaluation and prompt randomization script.
Table S4	Diagnostic Performance by Individual Intensivist Reviewer or Model
Table S5	Confusion Matrix Counts for Intensivist Reviewers and Models
Table S6	Group-Level Diagnostic Performance After Excluding Shared-Difficult Cases
Table S7	Quality-Stratified Mean Balanced Accuracy by Task and Assessor Group

Table S1. Model Identifiers, Access Routes, Evaluation Dates, and Settings

Display name	Exact model identifier	Access route	Evaluation date	Settings
Gemini 3 Flash	google/gemini-3-flash-preview	OpenRouter API	January 7, 2026	Fresh HTTPS request/session for each image; no conversation history retained; temperature=0; max_tokens=1000; random seed logged per batch
GPT-5.1	openai/gpt-5.1	OpenRouter API	January 7, 2026	Fresh HTTPS request/session for each image; no conversation history retained; temperature=0; max_tokens=1000; random seed logged per batch
Grok 4.1	x-ai/grok-4.1-fast	OpenRouter API	January 7, 2026	Fresh HTTPS request/session for each image; no conversation history retained; temperature=0; max_tokens=1000; random seed logged per batch
Claude Opus 4	anthropic/claude-opus-4	OpenRouter API	January 7, 2026	Fresh HTTPS request/session for each image; no conversation history retained; temperature=0; max_tokens=1000; random seed logged per batch
MedGemma 1.5	MedGemma-1.5-4b-it	Google Colab via Hugging Face API	January 30, 2026	Fresh HTTPS request/session for each image; no conversation history retained; temperature=0; max_tokens=1000; random seed logged per batch

Note. Fresh HTTPS request/session handling indicates that no conversation history was retained between images. The shared prompt template used for model evaluation is provided in Table S2.

Table S2. Prompt Template Used for MLLM Evaluation

Prompt Text
Analyze this chest X-ray image carefully.<br><br>PATIENT CONTEXT:<br>- Patient Gender: {patient_gender}<br>- Expected Catheter Side: {catheter_side} side placement<br>- Case ID: {case_number}<br><br>Look for central venous catheters (CVCs) and signs of pneumothorax. Use the patient context to help guide your assessment. If there are two CVCs, you only assess the CVC on the side given by context. You MUST make definitive decisions for CVC-related questions - uncertainty is not allowed for these assessments.<br><br>Return your response as a JSON object with these exact fields:<br><br>{<br>"cvc_access_site": "",<br>"cvc_tip_position": "",<br>"pneumothorax_present": "",<br>"reasoning": ""<br>}<br><br>Questions (REQUIRED DEFINITIVE ANSWERS):<br>1. What is the CVC access site? (MUST answer: {access_site_option_1} or {access_site_option_2})<br>2. Is the CVC tip position acceptable? (MUST answer: {tip_position_option_1} or {tip_position_option_2})<br>3. Are there signs of pneumothorax? (MUST answer: {pneumothorax_option_1} or {pneumothorax_option_2})<br>4. Brief reasoning for your assessments, considering the expected {catheter_side} side placement (1-2 sentences)<br><br>IMPORTANT: For ALL questions 1-3, you must choose one of the specified options. Base your decisions on what you can observe, even if the image quality is challenging.<br><br>Respond with ONLY the JSON object.

Note. Placeholders were replaced for each image using patient sex, catheter side, and case ID. Answer option order was randomized across cases. The same prompt template was used for all evaluated MLLMs.

Figure S1. Schematic of the image evaluation and session management pipeline.

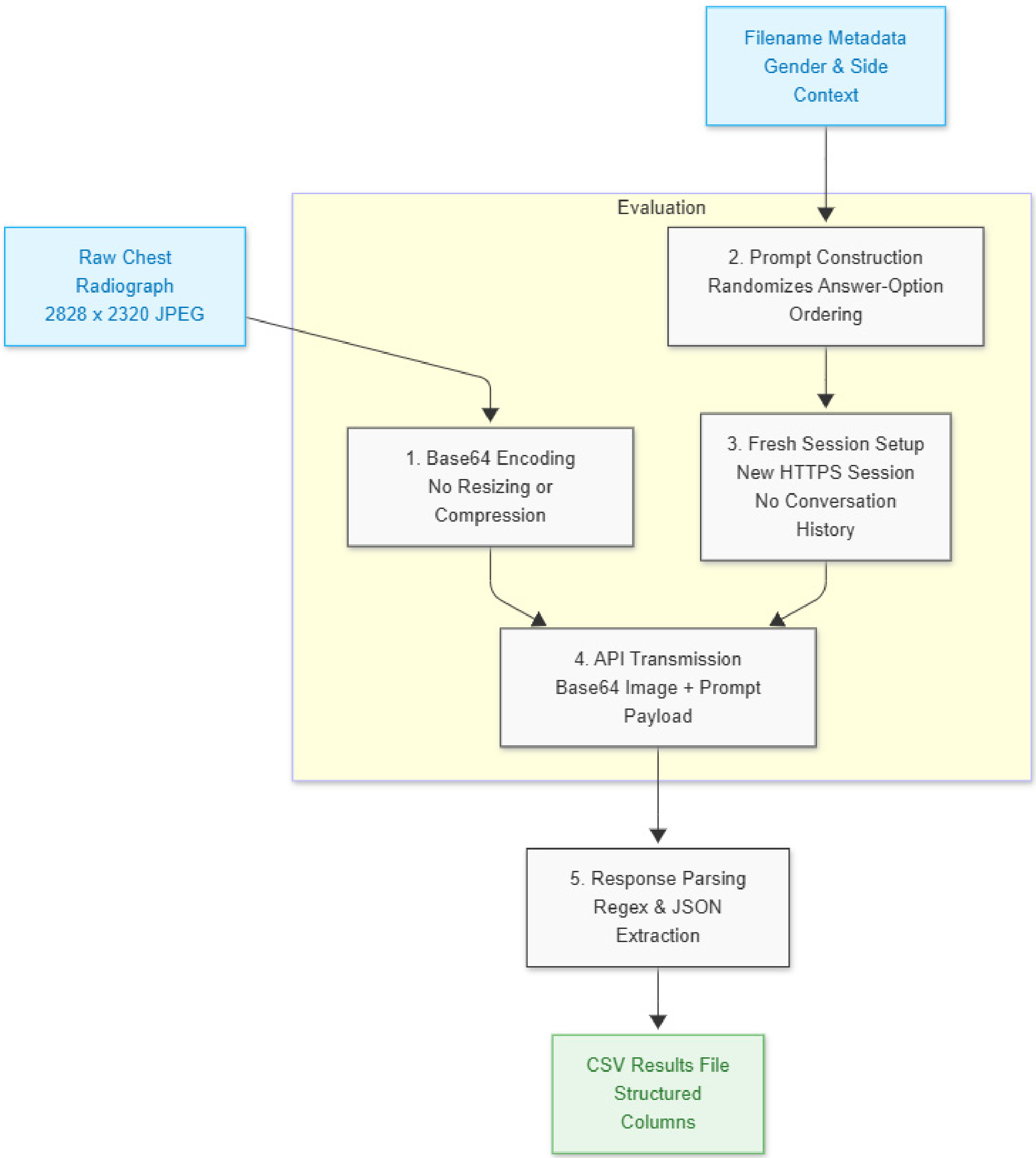

Table S3. Pseudocode of the image evaluation and prompt randomization script.

Pseudocode of the image evaluation and prompt randomization script.

```
Initialize API credentials and target Model ID
Load all radiograph image files from the input directory

For Each image file in directory:
    Extract metadata from filename:
        - Case Number (first 3 digits)
        - Patient Gender (M -> Male, F -> Female)
        - Expected Catheter Side (L -> Left, R -> Right)
    Store image file path and metadata in a list

Shuffle the order of the image list (prevents processing sequence bias)

For Each case in shuffled image list:
    1. PREPARE THE RANDOMIZED PROMPT:
       - Set Access Options = ["Jugular", "Subclavian"]
       - Set Tip Options = ["Acceptable", "Unacceptable"]
       - Set Pneumothorax Options = ["Present", "Absent"]

       - Randomly shuffle the order of elements in each options list
       - Render the text prompt using the patient context and shuffled options
       - Record the randomized option order for bias tracking

    2. ENCODE THE RADIOGRAPH:
       - Read the raw image file in binary mode
       - Encode the raw binary bytes directly into a Base64 string
       - (Note: No image cropping, resizing, or compression is applied)

    3. CONSTRUCT THE API PAYLOAD:
       - Bind the text prompt and the Base64 image string into the message payload
       - Set model temperature to 0

    4. EXECUTE INDEPENDENT API REQUEST:
       - Initialize a completely fresh, isolated HTTPS session
       - Add a unique request ID header
       - Record Start Time

       - Send HTTP POST request containing payload to the model API
       - Wait for model response (timeout at 60 seconds)

       - Record End Time and compute Response Duration

    5. PARSE AND SAVE OUTCOME:
       - Read text response from model
       - Extract the JSON block from the text response
       - Parse keys: CVC Access, CVC Tip Position, Pneumothorax Present, and Reasoning

       - Append results to CSV report, including:
           * Filename and patient metadata
           * Shuffled option orderings
           * Timestamp and API response duration
           * Categorical classifications and justification text

    6. TEMPORAL ISOLATION:
       - Pause execution for 3 seconds
```

Table S4. Diagnostic Performance by Individual Intensivist Reviewer or Model

Task	Group	Reviewer	Accuracy (95% CI)	Sensitivity/recall (95% CI)	Specificity (95% CI)	Precision (95% CI)	F1 score (95% CI)	Balanced accuracy (95% CI)	Cohen's kappa (95% CI)	MCC (95% CI)
CVC access	Human	R1	0.99 (0.97-1.00)	0.99 (0.98-1.00)	0.96 (0.86-1.00)	0.99 (0.98-1.00)	0.99 (0.98-1.00)	0.98 (0.93-1.00)	0.95 (0.86-1.00)	0.95 (0.86-1.00)
CVC access	Human	R2	0.99 (0.98-1.00)	0.99 (0.98-1.00)	1.00 (1.00-1.00)	1.00 (1.00-1.00)	1.00 (0.99-1.00)	1.00 (0.99-1.00)	0.98 (0.92-1.00)	0.98 (0.92-1.00)
CVC access	Human	R3	0.99 (0.98-1.00)	1.00 (1.00-1.00)	0.96 (0.86-1.00)	0.99 (0.98-1.00)	1.00 (0.99-1.00)	0.98 (0.93-1.00)	0.98 (0.92-1.00)	0.98 (0.92-1.00)
CVC access	Human	R4	0.96 (0.93-0.98)	1.00 (1.00-1.00)	0.71 (0.50-0.88)	0.96 (0.92-0.98)	0.98 (0.96-0.99)	0.85 (0.75-0.94)	0.81 (0.64-0.93)	0.82 (0.69-0.93)
CVC access	MLLM	Claude Opus 4	0.55 (0.48-0.62)	0.57 (0.50-0.65)	0.42 (0.21-0.63)	0.87 (0.80-0.93)	0.69 (0.62-0.75)	0.49 (0.39-0.61)	-0.01 (-0.12-0.11)	-0.01 (-0.15-0.14)
CVC access	MLLM	Gemini 3 Flash	0.87 (0.82-0.92)	0.97 (0.94-0.99)	0.25 (0.08-0.43)	0.90 (0.84-0.94)	0.93 (0.90-0.96)	0.61 (0.53-0.70)	0.28 (0.07-0.48)	0.31 (0.09-0.51)
CVC access	MLLM	GPT-5.1	0.61 (0.54-0.67)	0.64 (0.57-0.71)	0.38 (0.18-0.57)	0.87 (0.81-0.93)	0.74 (0.68-0.79)	0.51 (0.40-0.61)	0.01 (-0.10-0.12)	0.01 (-0.13-0.15)
CVC access	MLLM	Grok 4.1	0.53 (0.46-0.60)	0.55 (0.47-0.62)	0.42 (0.23-0.62)	0.86 (0.79-0.93)	0.67 (0.60-0.73)	0.48 (0.38-0.59)	-0.02 (-0.12-0.09)	-0.03 (-0.16-0.12)
CVC access	MLLM	MedGemma 1.5	0.34 (0.27-0.41)	0.26 (0.20-0.33)	0.88 (0.73-1.00)	0.93 (0.85-1.00)	0.40 (0.32-0.49)	0.57 (0.49-0.63)	0.04 (-0.01-0.10)	0.10 (-0.02-0.21)
CVC tip position	Human	R1	0.78 (0.72-0.84)	0.81 (0.74-0.88)	0.71 (0.59-0.83)	0.87 (0.80-0.93)	0.84 (0.79-0.89)	0.76 (0.69-0.83)	0.50 (0.36-0.63)	0.50 (0.37-0.63)
CVC tip position	Human	R2	0.81 (0.75-0.87)	0.77 (0.69-0.84)	0.93 (0.84-0.98)	0.96 (0.92-0.99)	0.85 (0.80-0.90)	0.85 (0.79-0.89)	0.61 (0.49-0.72)	0.64 (0.54-0.74)
CVC tip position	Human	R3	0.81 (0.75-0.86)	0.95 (0.91-0.98)	0.47 (0.33-0.61)	0.81 (0.74-0.87)	0.88 (0.83-0.91)	0.71 (0.65-0.79)	0.48 (0.34-0.62)	0.51 (0.37-0.65)
CVC tip position	Human	R4	0.78 (0.72-0.84)	0.95 (0.92-0.98)	0.38 (0.25-0.51)	0.78 (0.72-0.84)	0.86 (0.81-0.90)	0.67 (0.60-0.73)	0.39 (0.24-0.53)	0.43 (0.28-0.57)
CVC tip position	MLLM	Claude Opus 4	0.70 (0.63-0.77)	1.00 (1.00-1.00)	0.00 (0.00-0.00)	0.70 (0.63-0.77)	0.82 (0.78-0.87)	0.50 (0.50-0.50)	0.00 (0.00-0.00)	N/A (N/A-N/A)
CVC tip position	MLLM	Gemini 3 Flash	0.69 (0.63-0.76)	0.98 (0.96-1.00)	0.02 (0.00-0.06)	0.70 (0.63-0.77)	0.82 (0.77-0.86)	0.50 (0.48-0.53)	0.00 (-0.04-0.07)	0.01 (-0.10-0.17)
CVC tip position	MLLM	GPT-5.1	0.69 (0.62-0.75)	0.98 (0.95-1.00)	0.02 (0.00-0.06)	0.70 (0.63-0.76)	0.81 (0.76-0.86)	0.50 (0.48-0.52)	-0.01 (-0.06-0.06)	-0.02 (-0.12-0.14)
CVC tip position	MLLM	Grok 4.1	0.70 (0.63-0.77)	1.00 (1.00-1.00)	0.00 (0.00-0.00)	0.70 (0.63-0.77)	0.82 (0.78-0.87)	0.50 (0.50-0.50)	0.00 (0.00-0.00)	N/A (N/A-N/A)
CVC tip position	MLLM	MedGemma 1.5	0.70 (0.63-0.77)	1.00 (1.00-1.00)	0.00 (0.00-0.00)	0.70 (0.63-0.77)	0.82 (0.78-0.87)	0.50 (0.50-0.50)	0.00 (0.00-0.00)	N/A (N/A-N/A)
Pneumothorax findings	Human	R1	0.97 (0.94-0.99)	0.60 (0.00-1.00)	0.98 (0.96-1.00)	0.50 (0.00-1.00)	0.55 (0.22-0.86)	0.79 (0.49-0.99)	0.53 (-0.01-0.85)	0.53 (-0.01-0.86)
Pneumothorax findings	Human	R2	0.97 (0.94-0.99)	0.80 (0.33-1.00)	0.98 (0.95-0.99)	0.50 (0.14-0.85)	0.61 (0.25-0.89)	0.89 (0.66-1.00)	0.60 (0.21-0.88)	0.62 (0.25-0.88)
Pneumothorax findings	Human	R3	0.96 (0.93-0.99)	0.60 (0.00-1.00)	0.97 (0.94-0.99)	0.38 (0.00-0.75)	0.46 (0.17-0.77)	0.79 (0.49-0.99)	0.44 (-0.01-0.76)	0.46 (-0.02-0.77)
Pneumothorax findings	Human	R4	0.98 (0.96-0.99)	0.60 (0.00-1.00)	0.99 (0.97-1.00)	0.60 (0.00-1.00)	0.60 (0.25-0.92)	0.79 (0.49-1.00)	0.59 (-0.01-0.91)	0.59 (-0.01-0.91)
Pneumothorax findings	MLLM	Claude Opus 4	0.97 (0.94-0.99)	0.00 (0.00-0.00)	1.00 (1.00-1.00)	N/A (N/A-N/A)	N/A (N/A-N/A)	0.50 (0.50-0.50)	0.00 (0.00-0.00)	N/A (N/A-N/A)
Pneumothorax findings	MLLM	Gemini 3 Flash	0.97 (0.94-0.99)	0.00 (0.00-0.00)	1.00 (1.00-1.00)	N/A (N/A-N/A)	N/A (N/A-N/A)	0.50 (0.50-0.50)	0.00 (0.00-0.00)	N/A (N/A-N/A)
Pneumothorax findings	MLLM	GPT-5.1	0.97 (0.94-0.99)	0.00 (0.00-0.00)	1.00 (1.00-1.00)	N/A (N/A-N/A)	N/A (N/A-N/A)	0.50 (0.50-0.50)	0.00 (0.00-0.00)	N/A (N/A-N/A)
Pneumothorax findings	MLLM	Grok 4.1	0.97 (0.94-0.99)	0.00 (0.00-0.00)	1.00 (1.00-1.00)	N/A (N/A-N/A)	N/A (N/A-N/A)	0.50 (0.50-0.50)	0.00 (0.00-0.00)	N/A (N/A-N/A)
Pneumothorax findings	MLLM	MedGemma 1.5	0.97 (0.94-0.99)	0.00 (0.00-0.00)	1.00 (1.00-1.00)	N/A (N/A-N/A)	N/A (N/A-N/A)	0.50 (0.50-0.50)	0.00 (0.00-0.00)	N/A (N/A-N/A)

Note. Confidence intervals were estimated using case-resampling bootstrap with 2,000 replicates. R1-R4= human intensivist reviewers; MCC = Matthews correlation coefficient; MLLM = multimodal large language model. Pneumothorax-related diagnostic-performance estimates should be interpreted as exploratory because only 5 reference-standard positive cases were present.

Table S5. Confusion Matrix Counts for Intensivist Reviewers and MLLMs

Task	Positive reference class	Negative reference class	Group	Reviewer/model	TP	TN	FP	FN	Total
CVC access	Jugular access	Subclavian access	Human	R1	158	23	1	1	183
CVC access	Jugular access	Subclavian access	Human	R2	158	24	0	1	183
CVC access	Jugular access	Subclavian access	Human	R3	159	23	1	0	183
CVC access	Jugular access	Subclavian access	Human	R4	159	17	7	0	183
CVC access	Jugular access	Subclavian access	MLLM	Claude Opus 4	91	10	14	68	183
CVC access	Jugular access	Subclavian access	MLLM	Gemini 3 Flash	154	6	18	5	183
CVC access	Jugular access	Subclavian access	MLLM	GPT-5.1	102	9	15	57	183
CVC access	Jugular access	Subclavian access	MLLM	Grok 4.1	87	10	14	72	183
CVC access	Jugular access	Subclavian access	MLLM	MedGemma 1.5	41	21	3	118	183
CVC tip position	Appropriate	Inappropriate	Human	R1	104	39	16	24	183
CVC tip position	Appropriate	Inappropriate	Human	R2	98	51	4	30	183
CVC tip position	Appropriate	Inappropriate	Human	R3	122	26	29	6	183
CVC tip position	Appropriate	Inappropriate	Human	R4	122	21	34	6	183
CVC tip position	Appropriate	Inappropriate	MLLM	Claude Opus 4	128	0	55	0	183
CVC tip position	Appropriate	Inappropriate	MLLM	Gemini 3 Flash	126	1	54	2	183
CVC tip position	Appropriate	Inappropriate	MLLM	GPT-5.1	125	1	54	3	183
CVC tip position	Appropriate	Inappropriate	MLLM	Grok 4.1	128	0	55	0	183
CVC tip position	Appropriate	Inappropriate	MLLM	MedGemma 1.5	128	0	55	0	183
Pneumothorax-related findings	Findings present	Findings absent	Human	R1	3	175	3	2	183
Pneumothorax-related findings	Findings present	Findings absent	Human	R2	4	174	4	1	183
Pneumothorax-related findings	Findings present	Findings absent	Human	R3	3	173	5	2	183
Pneumothorax-related findings	Findings present	Findings absent	Human	R4	3	176	2	2	183
Pneumothorax-related findings	Findings present	Findings absent	MLLM	Claude Opus 4	0	178	0	5	183
Pneumothorax-related findings	Findings present	Findings absent	MLLM	Gemini 3 Flash	0	178	0	5	183
Pneumothorax-related findings	Findings present	Findings absent	MLLM	GPT-5.1	0	178	0	5	183
Pneumothorax-related findings	Findings present	Findings absent	MLLM	Grok 4.1	0	178	0	5	183
Pneumothorax-related findings	Findings present	Findings absent	MLLM	MedGemma 1.5	0	178	0	5	183

Note. Confusion matrix counts are shown for each human reviewer or MLLM against the three-radiologist consensus reference standard. Positive classes were jugular access for CVC access classification, appropriate CVC tip position for CVC tip assessment, and presence of pneumothorax-related radiographic findings for the pneumothorax task. TP = true positive; TN = true negative; FP = false positive; FN = false negative; CVC = central venous catheter; MLLM = multimodal large language model.

Table S6. Group-Level Diagnostic Performance After Excluding Shared-Difficult Cases

Task	Shared-difficult cases excluded	Cases analyzed	Group	Cohen's kappa	Accuracy	Sensitivity	Specificity	Precision	F1 score	Balanced accuracy	MCC	Fleiss kappa	Gwet AC1
CVC tip position	13	170	Human reviewers	0.585	0.840	0.871	0.744	0.920	0.890	0.808	0.609	0.444	0.623
CVC tip position	13	170	MLLMs	0.002	0.749	0.992	0.010	0.753	0.856	0.501	0.014	-0.008	0.983

Note. Values are group means across reviewers or models after excluding cases that were difficult for both humans and MLLMs, defined as cases for which at least 3 of 4 human reviewers and at least 3 of 5 MLLMs disagreed with the expert reference standard. This subgroup table is limited-CVC tip-position assessment because this was the only task for which excluding shared-difficult cases changed the analytic cohort. No shared-difficult cases were identified for CVC access classification, so no additional subgroup analysis was performed for that task. Pneumothorax-related findings were not analyzed in this subgroup because only 5 reference-standard positive cases were present, making subsetting unstable. MCC = Matthews correlation coefficient; MLLM = multimodal large language model.

Table S7. Quality-stratified Mean Balanced Accuracy by Task and Assessor Group

Task	Group	Good-quality radiographs	Poor-quality radiographs	Poor minus good
CVC access	Human reviewers	0.946	0.964	0.018
CVC access	MLLMs	0.538	0.519	-0.019
CVC tip position	Human reviewers	0.748	0.749	0.001
CVC tip position	MLLMs	0.499	0.502	0.003
Pneumothorax-related findings	Human reviewers	0.773	0.986	0.213
Pneumothorax-related findings	MLLMs	0.500	0.500	0.000

Note. Values are mean balanced accuracy across human reviewers (intensivists) or MLLMs within each radiograph-quality stratum. Radiographs with technical quality scores of 3 or 4 were classified as good quality, and those with scores of 0-2 were classified as poor quality. The poor-minus-good column is descriptive and was not used for formal hypothesis testing. Pneumothorax-related quality-stratified analyses are exploratory because positive cases were sparse after stratification. MLLM = multimodal large language model; CVC = central venous catheter.
