# Supplementary material for: Diagnostic Performance of Multimodal Large Language Models for Central Venous Catheter Assessment Chest Radiographs in the Intensive Care Unit
Source: Med Sci (Basel). 2026 Jun 14;14(2):315. doi: 10.3390/medsci14020315 (PMC13302893; doi:10.3390/medsci14020315)
Supplement: Supplementary file 1 [file medsci-14-00315-s001.zip › Supplementary_Material_S2.pdf]

Supplementary Material S2

Examples of Radiograph Cases with Responses

Comparison of Human Reviewers (R1-R4), Consensus Reference Standard (R5), and MLLMs

Title

Diagnostic Performance of Multimodal Large Language Models for Central Venous Catheter Assessment Chest Radiographs in the Intensive Care Unit

Abbreviations and Notation:

- CVC= Central Venous Catheter
- MLLM= Multimodal Large Language Model
- CVC Access: J = Jugular, S = Subclavian
- CVC Tip Position: Y = Appropriate CVC tip position, N = Inappropriate CVC tip position
- Pneumothorax: Y = Positive finding, N = No finding
- Normal font/ Green color indicates correct assessment relative to the consensus standard (R5).
- **Bold font**/ Red color indicates an incorrect assessment relative the consensus standard (R5).

Case 012MR - Jugular Access

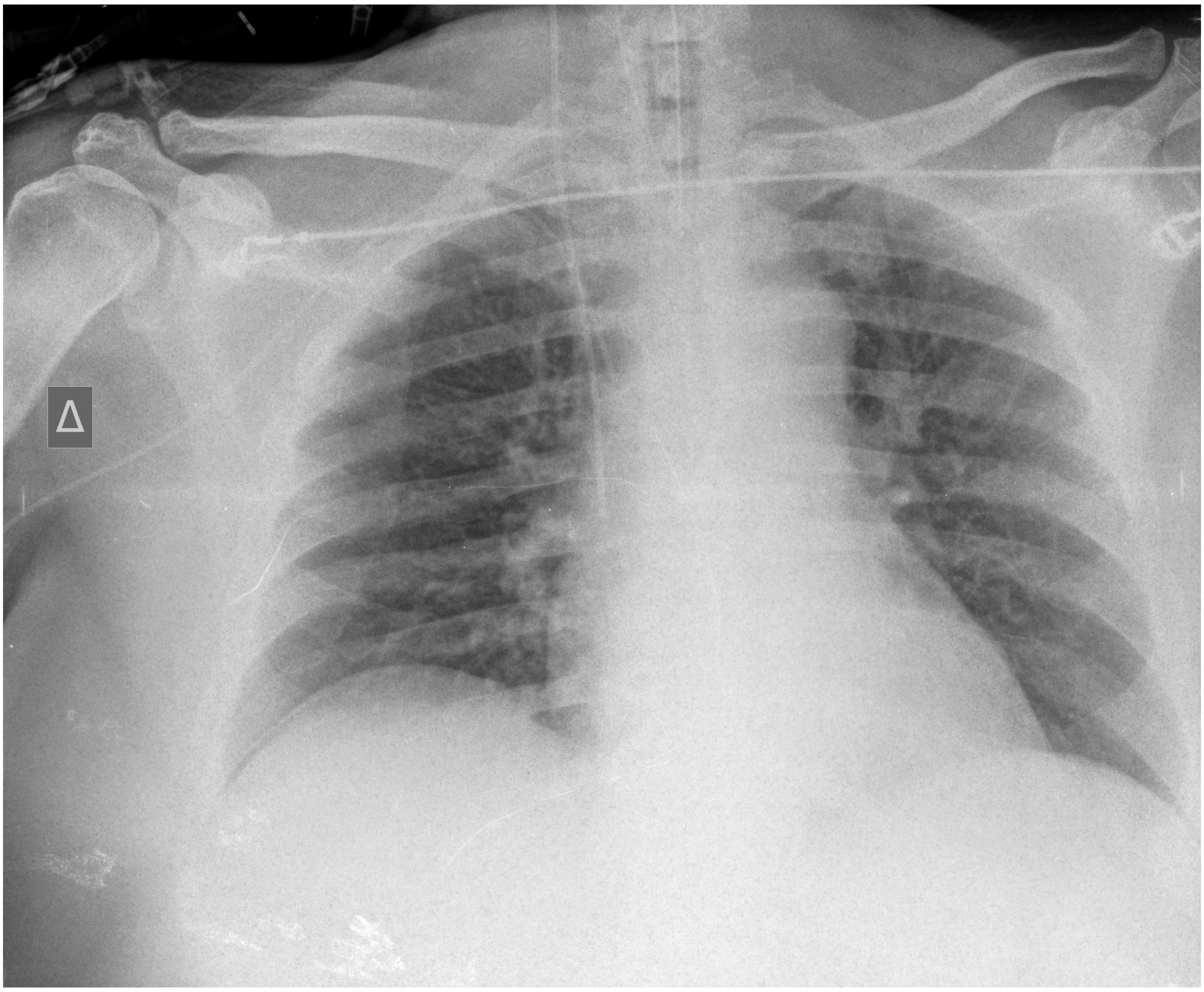

Assessor	Assessor Type	CVC Access	CVC Tip Position	Pneumothorax
**R5 (Consensus)**	**Reference Standard**	J	Y	N
R1	Human Reviewer	J	Y	N
R2	Human Reviewer	J	Y	N
R3	Human Reviewer	J	Y	N
R4	Human Reviewer	J	Y	N
Gemini 3 Flash	MLLM	J	Y	N
GPT-5.1	MLLM	J	Y	N
Claude Opus 4	MLLM	**S**	Y	N
Grok 4.1 Fast	MLLM	J	Y	N
MedGemma 1.5	MLLM	**S**	Y	N

Case 079MR - Subclavian Access

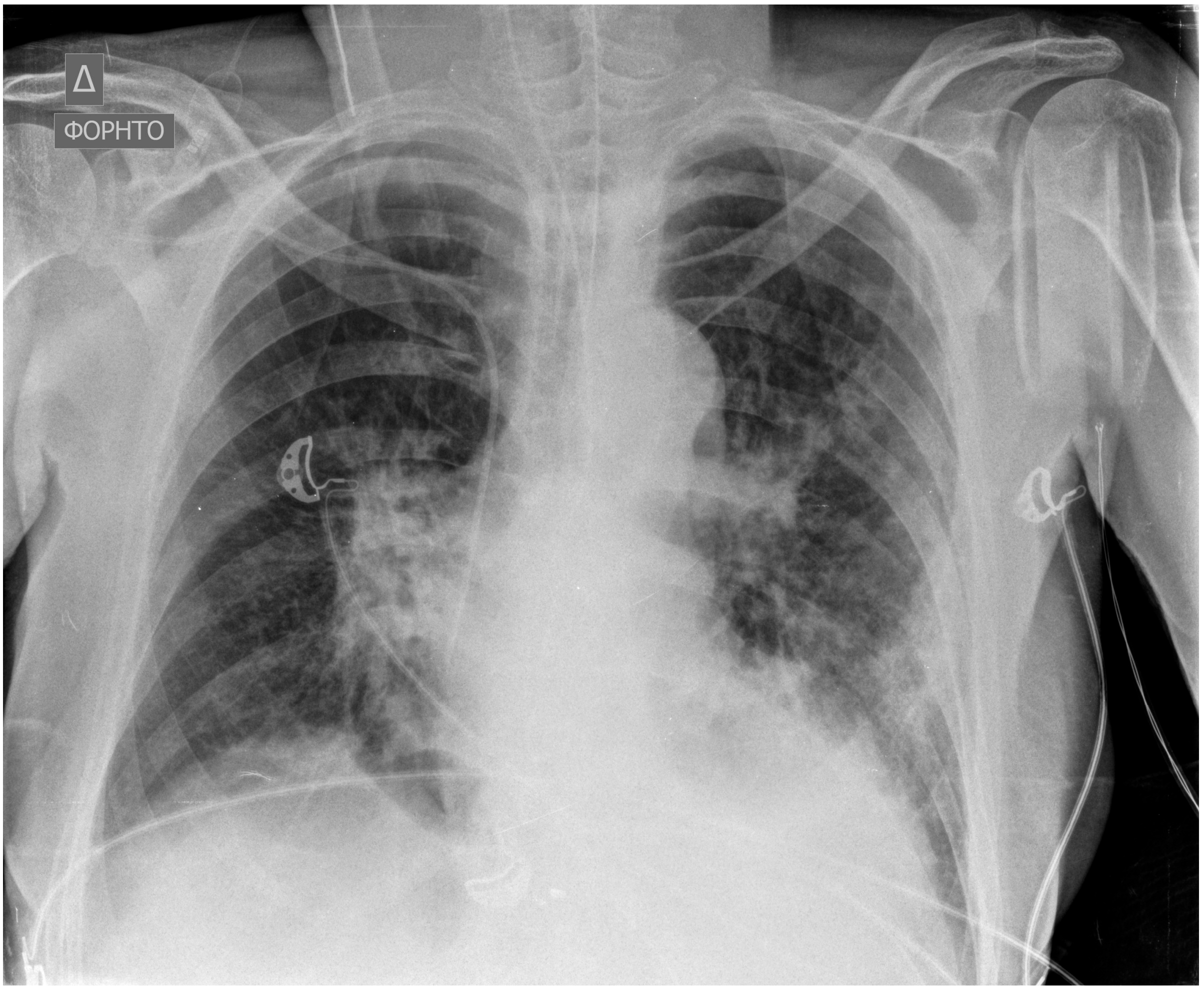

Assessor	Assessor Type	CVC Access	CVC Tip Position	Pneumothorax
**R5 (Consensus)**	**Reference Standard**	S	N	N
R1	Human Reviewer	S	N	N
R2	Human Reviewer	S	N	N
R3	Human Reviewer	S	N	N
R4	Human Reviewer	S	**Y**	N
Gemini 3 Flash	MLLM	**J**	**Y**	N
GPT-5.1	MLLM	S	**Y**	N
Claude Opus 4	MLLM	S	**Y**	N
Grok 4.1 Fast	MLLM	**J**	**Y**	N
MedGemma 1.5	MLLM	S	**Y**	N

Case 174ML - Jugular Access

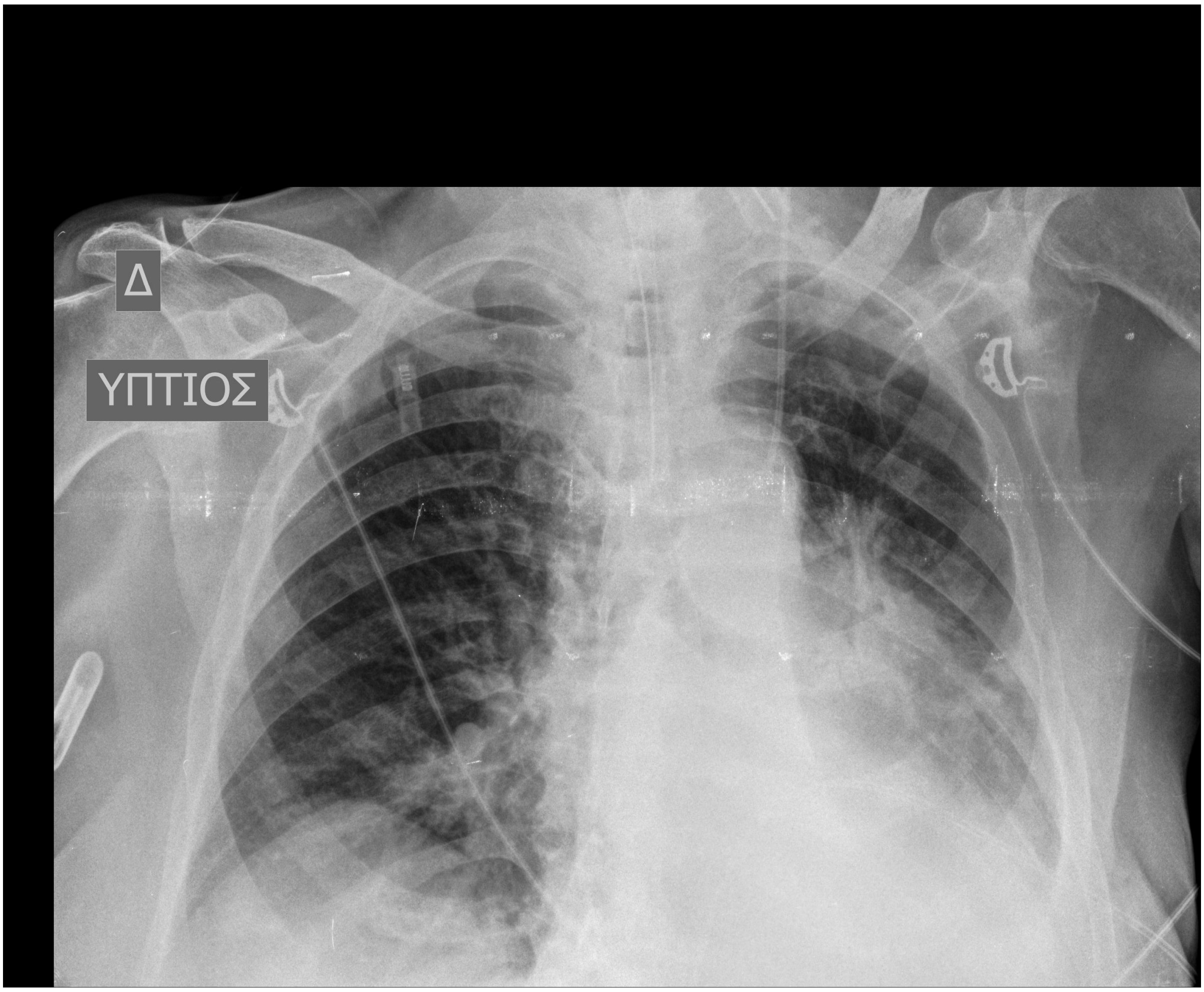

Assessor	Assessor Type	CVC Access	CVC Tip Position	Pneumothorax
**R5 (Consensus)**	**Reference Standard**	J	N	N
R1	Human Reviewer	J	N	N
R2	Human Reviewer	J	N	N
R3	Human Reviewer	J	N	N
R4	Human Reviewer	J	**Y**	N
Gemini 3 Flash	MLLM	J	**Y**	N
GPT-5.1	MLLM	**S**	**Y**	N
Claude Opus 4	MLLM	**S**	**Y**	N
Grok 4.1 Fast	MLLM	**S**	**Y**	N
MedGemma 1.5	MLLM	**S**	**Y**	N

Case 116MR - Jugular Access with Pneumothorax

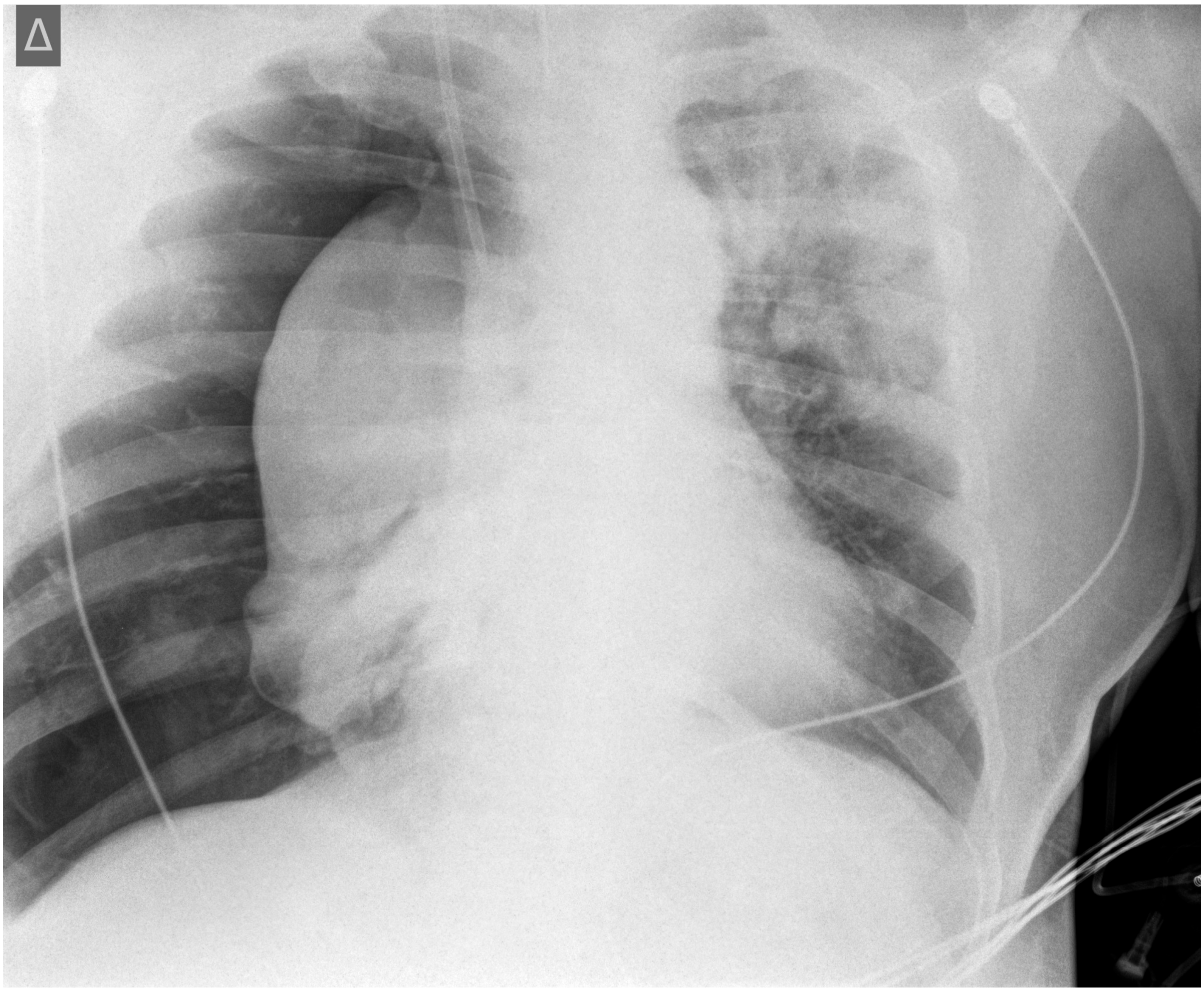

Assessor	Assessor Type	CVC Access	CVC Tip Position	Pneumothorax
**R5 (Consensus)**	**Reference Standard**	J	N	Y
R1	Human Reviewer	J	**Y**	Y
R2	Human Reviewer	J	N	Y
R3	Human Reviewer	J	N	Y
R4	Human Reviewer	J	**Y**	Y
Gemini 3 Flash	MLLM	J	**Y**	**N**
GPT-5.1	MLLM	**S**	**Y**	**N**
Claude Opus 4	MLLM	J	**Y**	**N**
Grok 4.1 Fast	MLLM	J	**Y**	**N**
MedGemma 1.5	MLLM	**S**	**Y**	**N**
